# Deep Molecular Characterization of Milder Spinal Muscular Atrophy Patients Carrying the c.859G>C Variant in *SMN2*

**DOI:** 10.3390/ijms23158289

**Published:** 2022-07-27

**Authors:** Laura Blasco-Pérez, Mar Costa-Roger, Jordi Leno-Colorado, Sara Bernal, Laura Alias, Marta Codina-Solà, Desirée Martínez-Cruz, Claudia Castiglioni, Enrico Bertini, Lorena Travaglini, José M. Millán, Elena Aller, Javier Sotoca, Raúl Juntas, Christina Engel Hoei-Hansen, Antonio Moreno-Escribano, Encarna Guillén-Navarro, Laura Costa-Comellas, Francina Munell, Susana Boronat, Ricardo Rojas-García, Mónica Povedano, Ivon Cuscó, Eduardo F. Tizzano

**Affiliations:** 1 Medicine Genetics Group, Vall d’Hebron Research Institute (VHIR), Universitat Autònoma de Barcelona (UAB), 08035 Barcelona, Spain; lblasco@vhebron.net (L.B.-P.); mar.costa@vhebron.net (M.C.-R.); jleno@vhebron.net (J.L.-C.); mcodina@vhebron.net (M.C.-S.); desiree.martinez@vhebron.net (D.M.-C.); icusco@santpau.cat (I.C.); 2 Department of Clinical and Molecular Genetics, Hospital Universitari Vall d’Hebron, Universitat Autònoma de Barcelona (UAB), 08035 Barcelona, Spain; 3 Genetics Department and Sant Pau Biomedical Research Institute, Hospital de la Santa Creu i Sant Pau, 08025 Barcelona, Spain; sbernal@santpau.cat (S.B.); lalias@santpau.cat (L.A.); 4 Centro de Investigación Biomédica en Red de Enfermedades Raras (CIBERER), 28029 Madrid, Spain; jose_millan@iislafe.es (J.M.M.); aller_ele@gva.es (E.A.); guillen.encarna@gmail.com (E.G.-N.); rrojas@santpau.cat (R.R.-G.); 5 Departamento de Neurología Pediátrica, Clínica Las Condes, 7591047 Santiago de Chile, Chile; castiglionic@gmail.com; 6 Unit of Neuromuscular and Neurodegenerative Disease, Ospedale Pediatrico Bambino Gesu, IRCCS, 00165 Rome, Italy; ebertini@gmail.com (E.B.); lorena.travaglini@opbg.net (L.T.); 7 Unidad de Genética, Hospital La Fe and IIS La Fe, 46026 Valencia, Spain; 8 Neuromuscular Diseases Unit, Neurology Department, Hospital Universitari Vall d’Hebron, 08035 Barcelona, Spain; jsotoca@vhebron.net (J.S.); rjuntas@vhebron.net (R.J.); 9 Department of Paediatrics, Copenhagen University Hospital, 2100 Copenhagen, Denmark; christina.hoei-hansen@regionh.dk; 10 Department of Clinical Medicine, University of Copenhagen, 1165 Copenhagen, Denmark; 11 Neurology Service and Medical Genetics Section, Hospital Clínico Universitario Virgen de la Arrixaca, IMIB-Arrixaca, Universidad de Murcia, 30120,Murcia, Spain; antonio_moreno9@yahoo.es; 12 Pediatric Neurology Section, Vall d’Hebron Research Institute (VHIR), Hospital Universitari Vall d’Hebron, Universitat Autònoma de Barcelona (UAB), 08035 Barcelona, Spain; laura.costa@vhebron.net (L.C.-C.); fmunell@vhebron.net (F.M.); 13 Pediatrics Department, Hospital de la Santa Creu i Sant Pau, 08025 Barcelona, Spain; sboronat@santpau.cat; 14 MND Clinic, Department of Neurology, Hospital de la Santa Creu i Sant Pau, Universitat Autònoma de Barcelona, 08025 Barcelona, Spain; 15 Unidad Funcional de Enfermedad de Motoneurona, Servicio de Neurología, Hospital Universitario de Bellvitge, 08907 Barcelona, Spain; 30058mpp@gmail.com

**Keywords:** spinal muscular atrophy, *SMN2* copies, phenotype–genotype correlations, positive modifiers, next-generation sequencing

## Abstract

Spinal muscular atrophy (SMA) is a severe neuromuscular disorder caused by biallelic loss or pathogenic variants in the *SMN1* gene. Copy number and modifier intragenic variants in *SMN2*, an almost identical paralog gene of *SMN1*, are known to influence the amount of complete SMN proteins. Therefore, *SMN2* is considered the main phenotypic modifier of SMA, although genotype–phenotype correlation is not absolute. We present eleven unrelated SMA patients with milder phenotypes carrying the c.859G>C-positive modifier variant in *SMN2*. All were studied by a specific NGS method to allow a deep characterization of the entire *SMN* region. Analysis of two homozygous cases for the variant allowed us to identify a specific haplotype, *Smn2-859C.1,* in association with c.859G>C. Two other cases with the c.859G>C variant in their two *SMN2* copies showed a second haplotype, *Smn2-859C.2,* in cis with *Smn2-859C.1*, assembling a more complex allele. We also identified a previously unreported variant in intron 2a exclusively linked to the *Smn2-859C.1* haplotype (c.154-1141G>A), further suggesting that this region has been ancestrally conserved. The deep molecular characterization of *SMN2* in our cohort highlights the importance of testing c.859G>C, as well as accurately assessing the *SMN2* region in SMA patients to gain insight into the complex genotype–phenotype correlations and improve prognostic outcomes.

## 1. Introduction

Spinal muscular atrophy (SMA) is a neuromuscular disorder characterized by the degeneration and loss of alpha motor neurons in the spinal cord anterior horns, leading to progressive atrophy of proximal muscles, weakness, respiratory failure, and even death. It is the second most common recessive genetic disease of infancy and early childhood with an incidence around 1:11,000 live births and a carrier frequency of 1:51 worldwide [1,2].

SMA patients are mainly classified into five clinical groups on the basis of age of onset, achieved motor milestones, and clinical severity. Type 0 or congenital, the most severe, appears prenatally, and the patient’s life expectancy is very short, usually a few weeks or months. Patients with type I, or Werdnig–Hoffmann disease (onset within the first six months of life), are never able to sit unsupported and generally do not survive beyond the age of two years. In the intermediate SMA type II (onset between 6 and 18 months of life), children acquire the ability to sit unsupported, but they never walk unaided and usually reach adolescence. Type III patients (Kugelberg–Welander disease) walk independently for a long time but eventually become wheelchair-bound. They can be further subdivided into type IIIa and IIIb depending on the age of disease onset (before or after three years of age). Finally, patients with SMA type IV present an adult onset and milder disease course [3,4,5]. It is important to bear in mind that current SMA therapies can modify the trajectory of SMA patients; therefore, this classification is mainly applied on clinical data prior to treatment [6,7].

At the molecular level, SMA is caused by the loss or mutation of both copies of the *survival of motor neuron 1 (SMN1)* gene, which encodes the survival motor neuron protein (SMN). In most cases, the disease is due to the homozygous absence of *SMN1* (95%), although pathogenic point variants have also been described [8,9,10].

Adjacent to *SMN1*, in a more centromeric position, lies *SMN2*, an almost identical paralog gene generated by a segmental duplication [11]. The fact that *SMN2* is present in humans and not in any other species suggests that the duplication of *SMN1* occurred recently in time. Consequently, the homology between both genes is extremely high, differing only in 16 positions called paralogous sequence variants (PSVs) [12,13]. This makes the region highly unstable, which leads to genomic instability predisposing to gene deletions, duplications, and conversions between both genes. Indeed, *SMN1* and *SMN2* genes can be present in multiple copies in the general population, both in cis and trans configuration [14].

Theoretically, the *SMN2* gene encodes the same protein as *SMN1,* but one of the PSVs, a silent transition in exon 7, alters the splicing pattern in most *SMN2* pre-mRNA transcripts. This causes the skipping of exon 7, resulting in a non-functional protein (SMN-Δ7) instead of the full-length protein [13]. As SMN-Δ7 is highly unstable and rapidly degraded, it is unable to compensate the absence or deficiency of *SMN1* in SMA patients [15]. It has been reported that each copy of *SMN2* can only produce about 10–15% of functional SMN proteins [16,17,18], being the number of *SMN2* copies the main modifier of SMA disease described to date.

Concretely, an inverse correlation between the number of *SMN2* copies and the severity of the phenotype has been widely reported, given that the higher the number of *SMN2* copies producing SMN functional protein, the milder the SMA phenotype [19,20,21]. Nevertheless, this correlation is not absolute, since discordant patients have been described in the literature, further classified as better-than-expected or worse-than-expected phenotypes according to their *SMN2* copy number [19,21]. It is known that the presence of the c.859G>C and c.835-44A>G (-44G) variants, located in exon 7 and intron 6 of *SMN2*, respectively, explains some of the better-than-expected discordant phenotypes. These SNVs, considered positive modifiers of SMA disease, increase the inclusion of exon 7 and therefore generate greater amounts of functional SMN protein [21,22,23,24,25].

The full characterization of *SMN2*, including dosage and structure, will be more relevant in the current scenario where new therapies for SMA are being implemented. It is well known that *SMN2* dosage is the main modifier of SMA, but it seems that this could be just the tip of the iceberg of a much more complicated framework. Indeed, all differences between *SMN1* and *SMN2* can be revealed by specific NGS studies [12]. It is also possible that these findings may relate to phenotype variability or to *SMN2*-specific treatment response [20].

In this work, we performed an in-depth characterization of the *SMN* region in eleven SMA patients carrying the c.859G>C modifier variant in the *SMN2* gene (*SMN2*^859C^) and presenting a milder phenotype. By defining the genetic background of *SMN2^859C^*, we discovered the existence of a common haplotype alongside the *SMN2* gene in linkage disequilibrium with the variant and a second less common haplotype harboring two *SMN2*^859C^ copies in cis.

## 2. Results

### 2.1. Clinical and Molecular Characterization of Patients

All SMA patients described in this study (ten males and one female) presented a biallelic absence of *SMN1* as the determinant of SMA and shared the presence of at least one copy of *SMN2*^859C^. Seven of these individuals carried two *SMN2* copies, including five with the c.859G>C modifier variant in their two *SMN2* genes (patients 1 to 5) and two with the variant in only one *SMN2* (patients 6 and 7). The other four patients presented three *SMN2* copies, and the variant was only present in one of their *SMN2* alleles (patients 8 to 11). A summary of the clinical and molecular data of the patients is shown in Table 1. Our cohort comprised SMA patients of Spanish, Italian, Danish, and Chilean origins, and the majority were classified as SMA type IIIb (8/11) and the remaining patients as SMA type IIIa (2/11). The remaining case (Patient 6 in Table 1) was classified as type II based on his age of onset, which was prior to 18 months. Currently, at three years of age, he has not yet achieved independent ambulation.

### 2.2. Haplotype Characterization by Deep Sequencing of SMN2 Genes

NGS data confirmed the biallelic absence of the entire *SMN1* gene in all patients, since specific nucleotides of *SMN2* were found in homozygous state (AB ratio of 100%) in all PSV positions. Similarly, the NGS results corroborated the *SMN2* copy number previously assigned by MLPA via the AB ratio analysis of all the different variants detected in the SMN region of each patient. In patients with two *SMN2* copies (except for patient 5), all variants were detected with an approximate allele frequency of 50% or 100%, whereas in patients with three *SMN2* copies, variants were found at a frequency of around 33%, 66%, or 100% (data not shown, available upon request). Patient 5 was a special case where variants were observed at a frequency of around 33–66–100% in the 5′ region and around 50–100% frequency in the 3′ region. This phenomenon was due to the presence of two complete *SMN2* genes and a partial *SMN* gene comprising exons 1 to 6 (*SMN1/2*Δ7-8) (see Table 2). In addition, the AB analysis of all patients confirmed the copy number of the c.859G>C modifier in each case.

Overall, our 11 patients represented 16 alleles with the c.859G>C variant, including five cases with two *SMN2*^859C^ and the remainder with just one allele with the variant (Table 2).

#### 2.2.1. Establishment of Two Haplotypes Associated with the c.859G>C Modifier Variant

We initially performed an in-depth analysis of the complete SMN2 region in patients 1 and 2, who carried two SMN2^859C^ genes and had consanguineous parents. The studies revealed that both patients were completely homozygous for the entire studied region and identical between them. Thus, we were able to determine the specific SMN2 sequence associated with the c.859G>C modifier in their alleles, establishing a haplotype called Smn2-859C.1 (Table 2). Similarly, sequencing results in patient 3 revealed an almost identical sequence to Smn2-859C.1 in his two SMN2 genes, with the exception of one rare variant (69356349-A-G) with an allele frequency of ~50%. In contrast, patients 4 and 5, who also presented two SMN2^859C^ copies, showed several variants in only one of their SMN2^859C^ along the studied region. Nonetheless, it was possible to infer that one of their SMN2 genes matched the sequence of the Smn2-859C.1 haplotype. Interestingly, in both patients, it was possible to assume a second haplotype associated with the c.859G>C variant that we defined as Smn2-859C.2 (Table 2). Applying this preliminary information, the Smn2-859C.1 haplotype was also inferred in one of the SMN2 copies of the remaining patients (patients 6 to 11), with few discrepant positions in patients 7, 10, and 11 (see Table 2).

**Figure 1:**
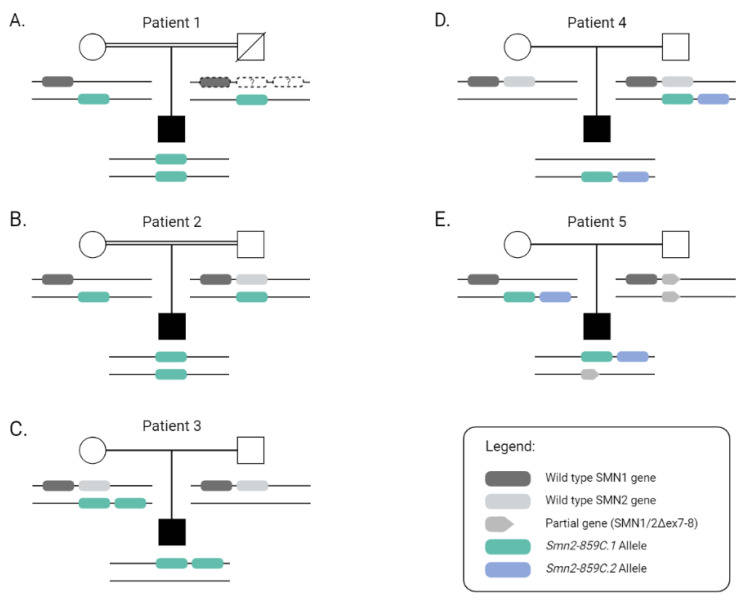
Pedigree representation of cases with two *SMN2*^859C^ copies. According to segregation studies, a cis or trans configuration was defined in each patient. (**A**) In patient 1, the father’s sample was not available, and *SMN2* configuration was inferred based on the results from the mother and patient. Given that the father did not present symptoms, we can assume that he carries at least one *SMN1* gene. In addition, being a consanguineous family, we assumed that *Smn2-859.C1* was transmitted by both parents (untested inferred alleles are represented by a dashed line). (**B**) In patient 2, the *Smn2-859.C1* haplotype was inherited from both parents, in agreement with the consanguinity in the family. (**C**) Patient 3 had two copies of *SMN2* with *Smn2-859.C1* in cis, inherited from his mother. (**D**) Patient 4 also had two copies of *SMN2* in cis, one with *Smn2-859.C1* and the other with *Smn2-859.C2* haplotype, forming a complex allele inherited from the father. (**E**) Patient 5 inherited the complex allele from his mother and the other allele with a partial non-functional *SMN1/2*Δ7-8 gene from his father.

To explore deeper into the structure of the *SMN2* genes, co-segregation studies from patients with two *SMN2*^859C^ were carried out through MLPA together with NGS or allele-specific PCR. These investigations showed that patient 2 carried his two *Smn2-859C.1* haplotypes in trans, inheriting one from each progenitor (Figure 1B). Patient 1′s co-segregation was incomplete, as a sample from his father was not available, but this family was consanguineous, and the mother only presented one *Smn2-859C.1* haplotype. Therefore, we could assume that his father also presented one *Smn2-859C.1* haplotype, and he should harbor both *Smn2-859C.1* haplotypes in trans (Figure 1A). In contrast, the co-segregation study in patient 3 revealed that both *Smn2-859C.1* haplotypes were in cis, forming a complex allele inherited from the mother (Figure 1C). Co-segregation in patients 4 and 5 indicated that the two *SMN2*^859C^ genes (*Smn2-859C.1* and *Smn2-859C.2* haplotypes) were located in cis. Specifically, patient 4 inherited this complex allele from his father and a null allele (without *SMN1* and *SMN2*) from his mother (Figure 1D), while patient 5 inherited the complex allele from his mother and the other allele with a partial non-functional *SMN1/2*Δ7-8 gene from his father (Figure 1E).

All together, these results indicated that the *Smn2-859C.1* haplotype was consistent in our cohort, since all patients presented it in association with the c.859G>C variant, either as a single allele or as part of a more complex allele formed by the *Smn2-859C.1* and *Smn2-859C.2* haplotypes in cis. Based on our 11 SMA patients, we have not observed any clinical difference between *Smn2-859C.1* and *Smn2-859C.2* haplotypes, although we only found two cases carrying the *Smn2-859C.2* haplotype.

#### 2.2.2. Difference between Haplotypes and Detection of a Novel Variant Exclusively Associated with the Smn2-859C.1 Haplotype

Analyzing the sequence of both haplotypes, *Smn2-859C.1* consists of 24 variants while *Smn2-859C.2* comprises 22 variants, sharing 16 of these positions and differing in the other 14. In particular, the sequence near the c.859G>C variant is shared between *Smn2-859C.1* and *Smn2-859C.2* haplotypes and spans at least 8848 bp (chr5:69365217-69374064). These haplotypes were not found in a total of 338 SMA patients without the c.859G>C variant, although some of the variants contained in the haplotypes are present in this larger cohort. Interestingly, we noticed the presence of a novel variant, c.154-1141G>A (69360651-G-A, hg19/GRCh37), located in intron 2a (Table 2). This variant was detected in all patients with the *Smn2-859C.1* haplotype but absent in the *Smn2-859C.2* haplotype. Moreover, this variant was not detected in the 338 SMA patients without the c.859G>C variant. The c.154-1141G>A change has not been reported in the general population according to gnomAD, ISB Kaviar3, and Bravo (as of 18 July 2022) [26,27,28]. In silico analysis of this deep intronic variant using the software SpliceAI [29], Alamut Visual Software version 2.11 (SOPHiA GENETICS), and ESRseq [30] did not predict an effect on the splicing process.

## 3. Discussion

Here, we present 11 patients with a clinical and molecular diagnosis of SMA caused by the biallelic absence of *SMN1* and with a milder phenotype explained by at least one *SMN2*^859C^ gene, given that 10 out of 11 patients were walkers. We identified a specific sequence, named *Smn2-859C.1,* present in all patients from our cohort in linkage disequilibrium with the c.859G>C variant. In addition, two cases showed a more complex allele, assembled by *Smn2-859C.1* and *Smn2-859C.2* in cis.

**Figure 2:**
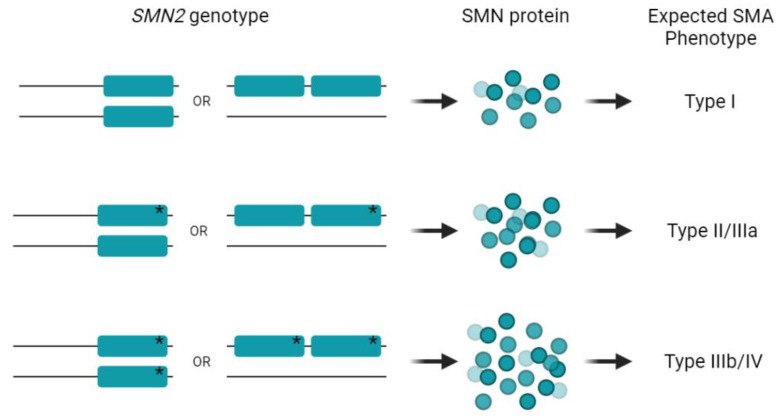
Expected SMA phenotype in cases with two *SMN2* copies according to the presence of c.859G>C. An additive effect on SMA phenotype is observed depending on whether the c.859G>C variant is found in one or both *SMN2* copies. *SMN2* gene is represented as a rectangle, and the presence of the c.859G>C variant in exon 7 is indicated by an asterisk. Not all *SMN2* genotypes represented in this figure were detected in this study (see Figure 1 for more details).

In order to study the genetic origin of the c.859G>C variant, we expanded our cohort of Spanish cases with patients from Denmark, Italy, and Chile. We applied NGS methodologies exclusively focused on the SMN region to determine the exact sequence of *SMN2* associated with the c.859G>C variant in each patient [12]. By studying the patients with two *SMN2*^859C^, we were able to determine two haplotypes associated with the variant, *Smn2-859C.1* and *Smn2-859C.2*. The *Smn2-859C.1* haplotype, with minor modifications, was present in all 11 patients (14/16 *SMN2*^859C^ alleles), either in cis or trans configuration, while the *Smn2-859C.2* haplotype was only found in two patients (2/16 *SMN2*^859C^ alleles), always in cis configuration with the *Smn2-859C.1* haplotype (Figure 2). Notably, no patient was found to harbor the c.859G>C variant in association with any other haplotype, regardless of their ethnic lineage, which points towards a common ancestral origin in all cases.

The c.859G>C variant has been previously reported to increase the inclusion of *SMN2* exon 7 by 20%, which leads to the generation of higher amounts of functional proteins than the wild-type *SMN2* gene [22,25]. Patients carrying this variant developed milder SMA phenotypes compared with those with the same *SMN2* copy number but without the variant [23]. In our case, the deep characterization of the entire SMN region supports that *SMN2*^859C^ is, at first sight, primarily responsible for the milder phenotype in our patients.

To date, together with our six newly described patients, a total of 44 patients carrying c.859G>C have been reported worldwide, including a patient recently detected by newborn screening [21,22,23,25,31,32,33,34]. In general population databases, the c.859G>C variant is reported at a frequency of approximately 0.3% with 132 homozygotes detected [26]. However, it is possible that the data are not accurate given the high homology between *SMN1* and *SMN2* and their copy number variability, which poses a challenge in the analysis and proper annotation of the SMN region with non-specific NGS techniques, such as exome or genome. Nevertheless, it is possible to estimate the frequency of this variant in the SMA population based on previous studies. According to the data of Calucho et al. (2018) [19], the allelic frequency of the c.859G>C variant is 1.04% (13/1250 alleles) in a series of 625 Spanish SMA patients. In fact, in this cohort, approximately 25% of better-than-expected cases with two *SMN2* copies carried the variant [19]. Although c.859G>C appears to be relatively uncommon, at present it is not routinely tested in SMA patients, deserving more studies to clearly establish its incidence.

Concerning clinical classification, patients with two *SMN2* genes usually debut in the first six months of life and are classified as SMA type I [19]. In our series, patients with two *SMN2* copies and the positive modifier presented at least type II or type III disease (Table 1). Furthermore, an additive effect was observed since patients with the c.859G>C change in both *SMN2* genes had a better phenotype than patients carrying the variant only in one *SMN2*, confirming previous observations [23] (Figure 2). For instance, patient 3 (with two *SMN2*^859C^ copies) developed the first SMA symptoms at 12 years of age, being classified as type IIIb, whereas patient 7 (with the variant in one of his *SMN2* genes) had manifestations at 18 months of life with a clinical diagnosis of type IIIa. Regarding cases with three *SMN2* copies, all patients presented the c.859G>C variant in only one of their alleles, developing a type III phenotype. Interestingly, we did not find any patient with three *SMN2* copies and the variant in more than one allele and, in fact, no patient with this genotype has been described in the literature either. This could be due to the fact that patients with three *SMN2* copies showing SMA type II or III are not currently tested for the variant. Another reason could be that cases with this genotype perhaps do not manifest clear disease symptoms due to the higher production of SMN protein and therefore may never be diagnosed. Similarly, it has been previously speculated that some individuals with zero *SMN1* and four or five *SMN2* copies may present minimal symptoms or be asymptomatic throughout their lives, remaining undetected [35]. This corroborates the importance of implementing detection of the c.859G>C-positive modifier as part of the genetic diagnosis routine in SMA.

At this level of analysis and based on the clinical information available for each patient, we did not observe categorical phenotypic differences between the *Smn2-859C.1* or *Smn2-859C.2* haplotypes, nor the cis or trans configuration of the *Smn2-859C.1* haplotype, since all cases with two *SMN2*^859C^ copies presented a milder phenotype (IIIb). Interestingly, patient 2, with the exact same sequence and configuration as patient 1, also developed type IIIb SMA, but his onset was noted earlier in comparison with patient 1 and the remaining cases with two *SMN2*^859C^. At present, we are unable to explain this minor disparity considering all the studies performed in *SMN2*. Thus, this fact suggests disease onset could also be conditioned by as yet unknown factors, other than *SMN2* structure.

**Figure 3:**
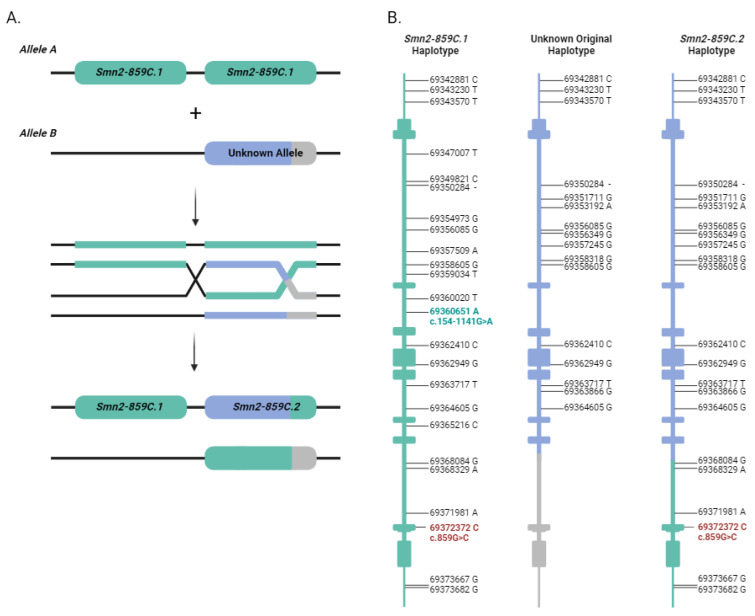
Proposed mechanism of origin and structure of *Smn2-859C.1* and *Smn2-859C.2* haplotypes. (**A**) Hypothetic origin of the *Smn2-859C.2* haplotype through homologous recombination involving a double cross-over event between allele A (with two *Smn2-859C.1* haplotypes represented in green) and allele B (containing the 5′ region of the *Smn2-859C.2* haplotype, in blue, and the 3’ end with an unknown sequence, in grey). (**B**) *SMN2* structure details (representing exons and introns from top to bottom) and location of variants of the *Smn2-859C.1* and *Smn2-859C.2* haplotypes, as well as the unknown original haplotype that presumably originated the *Smn2-859C.2* haplotype. The c.154-1141G>A (69360651-G-A, hg19/GRCh37) variant is indicated in green, whereas the c.859G>C modifier is shown in red (further explanation in the text and Table 2).

As mentioned above, the *Smn2-859C.2* haplotype was detected in cis configuration with respect to *Smn2-859C.1*, assembling a complex allele containing two different *SMN2*^859C^ genes (Figure 1). These two haplotypes differ in several positions, but an identical block of at least 8848 bp around c.859G>C is present in both (Table 2 and Figure 3B). This observation, together with the fact that we also detected an allele formed by two *Smn2-859C.1* haplotypes in cis, points towards a possible origin of the complex allele through homologous recombination, implicating a double cross-over event [36]. In this event, two alleles would be involved (Figure 3A): allele A, consisting of two *SMN2* genes with the *Smn2-859C.1* haplotype, and allele B, formed by at least one *SMN2* with an unknown haplotype containing part of *Smn2-859C.2* but without the c.859G>C variant. In the double homologous recombination process, allele A would maintain both c.859G>C variants as well as gain the part of the *Smn2-859C.2* haplotype from allele B, generating the complex allele that we detected in our patients (Figure 3B).

Finally, it should be noted that the *Smn2-859C.1* haplotype contains the novel variant c.154-1141G>A, located in intron 2a. According to our results, this variant is in linkage disequilibrium with the c.859G>C modifier given that, in our larger cohort of 349 SMA patients, it was only detected in those carrying the c.859G>C variant, and it was not found in population databases. This observation suggests that the sequence between this variant and the c.859G>C modifier has been ancestrally conserved. In silico splicing tools did not predict any specific effect of this deep intronic variant. However, we could not rule out some influence of this change, given the limitations of splicing predictors; thus, it deserves further investigation.

Our NGS approach to characterize these patients revealed new information that could be relevant for the different functions and/or alterations of *SMN2*. It is important to consider whether the function and expression of *SMN2* is not only modified depending on the cis or trans configuration of *SMN2*^859C^ but also on the presence of the *Smn2-859C.1* or *Smn2-859C.2* haplotype. Long regulators, cis- or trans-acting elements, may distinctively influence its function and/or expression according to the topography of the region.

## 4. Materials and Methods

### 4.1. Study Participants

We studied eleven unrelated SMA patients from different international centers with the presence of at least one *SMN2*^859C^ gene. Patients were classified into SMA type according to age of onset, clinical severity, and achieved motor milestones, prior to receiving any modifying therapies. Criteria for correlating phenotype with *SMN2* dosage were type I (non-sitters) with two *SMN2* copies, type II (sitters) with three *SMN2* copies, and type III (walkers) patients with three–four *SMN2* copies [19]. Based on this model, our patients with two *SMN2* copies were considered discordant, as none presented a type I SMA phenotype (Table 1).

All patients were selected from a larger cohort of 349 SMA patients, undergoing an NGS study of the *SMN* region [12], based on the presence of the c.859G>C variant. Four of the patients were previously described as carriers of this variant (patients 1, 8, and 11 [23] and patient 7 [12]).

DNA samples were extracted from peripheral blood using standard methods. Ethics approval was granted by the Clinical Research Ethics Committee of Hospital Vall d’Hebron (Comité de Ética de Investigación con Medicamentos del Hospital Universitari Vall d’Hebron (PR(AG)229/2018)). Written informed consent was obtained from all participants or their parents/legal caregivers.

### 4.2. SMN2 Genotyping and Haplotype Characterization

All patients were genetically confirmed as SMA cases via previously described methods that also included testing *SMN2* modifier variants [10,23,37]. A detailed molecular characterization of *SMN2* was carried out in all patients by a specific NGS sequencing method [12].

In addition, to detect the presence of the c.859G>C variant in some progenitor samples, two specific PCRs were designed to amplify exons 7 and 8 of genes *SMN1* and *SMN2*. The allele-specific PCR technique [38] was used to amplify both genes separately to ascertain in which gene the variant was present. Standard Sanger sequencing was performed with the PCR products, allowing us to detect the c.859G>C variant. These primers are also designed to study the c.835-44A>G variant. Primer sequences and PCR conditions are provided in Table S1.

## 5. Conclusions

This series of patients with milder phenotypes demonstrates the relevance of testing the c.859G>C variant in all SMA patients, with special consideration in cases with two or three *SMN2* copies in the context of neonatal screening. Indeed, the presence of this rare variant in an asymptomatic neonate may help to predict a better phenotype by natural history per se, regardless of the therapeutic option chosen. This is crucial in order to evaluate the effects of the approved therapies to unmask long-term benefits in treated patients. Given that not all discordant cases can be explained by this positive variant, it is necessary to further analyze the *SMN2* region by NGS to detect other reported candidate variants [24] and the presence of hybrid *SMN1-SMN2* structures [20], as well as to unravel novel phenotypic modifier variants. In the current therapeutic context, genetic studies in patients confirmed with biallelic *SMN1* absence or pathogenic variants should consider not only testing for *SMN2* copies but also investigating *SMN2* variants and structures as part of the integral characterization of patients receiving expensive and sometimes lifelong therapies.

## Figures and Tables

**Table 1:** Clinical and molecular data of patients. Information regarding general characteristics of patients, SMA phenotype, and *SMN1/2* genotypes.

Patient	1	2	3	4	5	6	7	8	9	10	11
**Origin**	Spanish	Spanish	Chilean	Italian	Spanish	Spanish	Danish	Spanish	Spanish	Spanish	Spanish
**Gender**	Male	Male	Male	Male	Male	Male	Female	Male	Male	Male	Male
**Age (years)**	49	35	21	17	28	3	8	71	54	57	47
**Consanguinity**	Yes	Yes	No	No	No	No	No	No	No	No	No
**Reported**	Bernal et al., 2010 (P2) [23]	Bernal et al., 2010 (P3) [23]	This work	This work	This work	This work	Blasco-Pérez et al., 2021 [12]	Bernal et al., 2010 (P4) [23]	This work	This work	Bernal et al., 2010 (P5) [23]
**SMA type**	IIIb	IIIb	IIIb	IIIb	IIIb	II	IIIa	IIIb	IIIb	IIIa	IIIb
**Age of onset**	10 years	4 years	12 years	13 years	15 years	12 months	18 months	14 years	9-10 years	24 months	13 years
**Walked unaided**	Yes	Yes	Yes	Yes	Yes	Not yet accomplished	Yes	Yes	Yes	Yes	Yes
**Wheelchair bound age**	41 years	23 years	No	No	No	Not applicable	No	49 years	No	22 years	37 years
***SMN1* copies**	0	0	0	0	0	0	0	0	0	0	0
***SMN2* copies**	2	2	2	2	2 *	2	2	3	3	3	3
**Presence of c.859G>C**	2/2	2/2	2/2	2/2	2/2	1/2	1/2	1/3	1/3	1/3	1/3

* In addition, presents a partial *SMN* gene comprising only exons 1 to 6 (*SMN1/2*Δ7-8) (see Figure 1).

**Table 2:** Haplotype characterization of SMN2 genes. Detail of the 30 positions comprising the *Smn2-859C.1* (green) and *Smn2-859C.2* (blue) haplotypes in our patients. Punctual discrepancies are represented in red. The novel variant c.154-1141G>A (69360651-G-A, hg19/GRCh37), exclusively associated with the *Smn2-859C.1* haplotype, is indicated in green in the first column. The c.859G>C modifier variant is marked in red. The remaining alleles of each patient not carrying the c.859G>C are represented in grey.

		Patient		1		2		3		4		5			6		7		8			9			10			11		
Location	hg19	*Smn2* *-859C.1*	*Smn2* *-859C.2*	1	1	1	1	1	1	1	2	1	2	-	1	-	1	-	1	-	-	1	-	-	1	-	-	1	-	-
	69342881-T-C	C	C	C	C	C	C	C	C	C	C	C	C	C	C	C	C	C	C	C	C	C	C/T	C/T	C	C	C	C	C/T	C/T
	69343230-C-T	T	T	T	T	T	T	T	T	T	T	T	T	T	T	T	T	T	T	T	T	T	T	T	T	T	T	T	T	T
	69343570-G-T	T	T	T	T	T	T	T	T	T	T	T	T	T	T	G	T	T	T	T	T	T	T	T	T	T	T	T	T/G	T/G
intron 1	69347007-C-T	T		T	T	T	T	T	T	T	C	T	C	C	T	C	C	C	T	C	C	T	C	C	T	C	C	T	C	C
	69349821-T-C	C		C	C	C	C	C	C	C	T	C	T	T	C	T	C	T	C	T	T	C	T	T	C	T	T	C	T	T
	69350284-A--	-	-	-	-	-	-	-	-	-	-	-	-	-	-	A	-	-	-	-	-	-	-	-	-	-	-	A	A	A
	69351711-A-G		G	A	A	A	A	A	A	A	G	A	G	A	A	A	A	A	A	A	A	A	A	A	A	A	A	A	A	A
	69353192-G-A		A	G	G	G	G	G	G	G	A	G	A	A	G	G	G	G	G	G	G	G	G	G	G	G	G	G	G	G
	69354973-A-G	G		G	G	G	G	G	G	G	A	G	A	G	G	A	G	G	G	G	G	G	G	G	G	G	G	G	G/A	G/A
	69356085-A-G	G	G	G	G	G	G	G	G	G	G	G	G	G	G	G	G	G	G	G	G	G	G	G	G	G	G	G	G	G
	69356349-A-G		G	A	A	A	A	A	G	A	G	A	G	A	A	A	A	A	A	A	A	A	A	A	A	A	A	A	A	A
	69357245-C-G		G	C	C	C	C	C	C	C	G	C	G	G	C	C	C	G	C	G	G	C	G	G	C	G	G	C	C/G	C/G
	69357509-G-A	A		A	A	A	A	A	A	A	G	A	G	G	A	G	A	G	A	G	G	A	G	G	A	G	G	A	G	G
	69358318-A-G		G	A	A	A	A	A	A	A	G	A	G	A	A	A	A	A	A	A	A	A	A	A	A	A	A	A	A	A
	69358605-A-G	G	G	G	G	G	G	G	G	G	G	G	G	G	G	A	G	G	G	G	G	G	G	G	G	G	G	G	G/A	G/A
	69359034-C-T	T		T	T	T	T	T	T	T	C	T	C	C	T	C	T	C	T	C	C	T	C	C	T	C	C	T	C	C
intron 2a	69360020-G-T	T		T	T	T	T	T	T	T	G	T	G	G	T	G	T	G	T	G	G	T	G	G	T	G	G	G	G	G
	**69360651-G-A**	A		A	A	A	A	A	A	A	G	A	G	G	A	G	A	G	A	G	G	A	G	G	A	G	G	A	G	G
intron 2b	69362410-T-C	C	C	C	C	C	C	C	C	C	C	C	C	T	C	T	C	T	C	T	T	C	T	T	C	T	T	C	T	T
exon 3	69362949-A-G	G	G	G	G	G	G	G	G	G	G	G	G	G	G	A	G	G	G	G	G	G	G	G	G	G	G	G	G/A	G/A
intron 4	69363717-C-T	T	T	T	T	T	T	T	T	T	T	T	T		T	C	T	C	T	C	C	T	C	C	C	C	C	T	C	C
	69363866-A-G		G	A	A	A	A	A	A	A	G	A	G		A	A	A	A	A	A	A	A	A	A	A	A	A	A	A	A
	69364605-A-G	G	G	G	G	G	G	G	G	G	G	G	G		G	A	G	G	G	G	G	G	G	G	G	G	G	G	G/A	G/A
intron 5	69365216-G-C	C		C	C	C	C	C	C	C	G	C	G		C	G	C	G	C	G	G	C	G	G	C	G	G	C	G	G
intron 6	69368084-A-G	G	G	G	G	G	G	G	G	G	G	G	G		G	A	G	G	G	G/A	G/A	G	G	G	G	G	G	G	G/A	G/A
	69368329-G-A	A	A	A	A	A	A	A	A	A	A	A	A		A	G	A	A	A	G/A	G/A	A	A	A	A	A	A	A	G/A	G/A
	69371981-C-A	A	A	A	A	A	A	A	A	A	A	A	A		A	C	A	A	A	A	A	A	A	A	A	A	A	A	A/C	A/C
exon 7	**69372372-G-C**	C	C	C	C	C	C	C	C	C	C	C	C		C	G	C	G	C	G	G	C	G	G	C	G	G	C	G	G
downstream	69373667-A-G	G	G	G	G	G	G	G	G	G	G	G	G		G	A	G	G	G	G	G	G	G	G	G	G	G	G	G	G
	69373682-C-G	G	G	G	G	G	G	G	G	G	G	G	G		G	C	G	C	G	C	C	G	C	C	G	C	C	G	G/C	G/C

## Data Availability

All data and scripts used to generate the analyses of this paper are available upon request, unless the type of request compromises ethical standards or legal requirements.

## Supplementary Materials

The following supporting information can be downloaded at: https://www.mdpi.com/article/10.3390/ijms23158289/s1.

## References

[1] Cusin V., Clermont O., Gérard B., Chantereau D., Elion J. (2003). Prevalence of SMN1 deletion and duplication in carrier and normal populations: Implication for genetic counselling. J. Med. Genet..

[2] Sugarman E.A., Nagan N., Zhu H., Akmaev V.R., Zhou Z., Rohlfs E.M., Flynn K., Hendrickson B.C., Scholl T., Sirko-Osadsa D.A. (2012). Pan-ethnic carrier screening and prenatal diagnosis for spinal muscular atrophy: Clinical laboratory analysis of >72 400 specimens. Eur. J. Hum. Genet..

[3] Zerres K., Rudnik-Schöneborn S. (1995). Natural history in proximal spinal muscular atrophy. Clinical analysis of 445 patients and suggestions for a modification of existing classifications. Arch Neurol..

[4] Zerres K., Wirth B., Rudnik-Schöneborn S. (1997). Spinal muscular atrophy clinical and genetic correlations. Neuromuscul. Disord..

[5] Wang C.H., Finkel R.S., Bertini E.S., Schroth M., Simonds A., Wong B., Aloysius A., Morrison L., Main M., Crawford T.O. (2007). Consensus statement for standard of care in spinal muscular atrophy. J. Child Neurol..

[6] Tizzano E.F., Finkel R.S. (2017). Spinal muscular atrophy: A changing phenotype beyond the clinical trials. Neuromuscul. Disord..

[7] Schorling D.C., Pechmann A., Kirschner J. (2020). Advances in Treatment of Spinal Muscular Atrophy – New Phenotypes, New Challenges, New Implications for Care. J. Neuromuscul. Dis..

[8] Lefebvre S., Bürglen L., Reboullet S., Clermont O., Burlet P., Viollet L., Benichou B., Cruaud C., Millasseau P., Zeviani M. (1995). Identification and characterization of a spinal muscular atrophy-determining gene. Cell.

[9] Burghes A.H.M., McGovern V.L. (2017). Genetics of spinal muscular atrophy. Molecular and Cellular Therapies for Motor Neuron Diseases.

[10] Alías L., Bernal S., Fuentes-Prior P., Barceló M.J., Also E., Martínez-Hernández R., Rodríguez-Alvarez F.J., Martín Y., Aller E., Grau E. (2009). Mutation update of spinal muscular atrophy in Spain: Molecular characterization of 745 unrelated patients and identification of four novel mutations in the SMN1 gene. Hum. Genet..

[11] Rochette C.F., Gilbert N., Simard L.R. (2001). SMN gene duplications and the emergence of the SMN2 gene ocurred in distinct hominids: SMN2 is unique to Homo sapiens. Hum. Genet..

[12] Blasco-Pérez L., Paramonov I., Leno J., Bernal S., Alias L., Fuentes-Prior P., Cuscó I., Tizzano E.F. (2021). Beyond copy number: A new, rapid, and versatile method for sequencing the entire SMN2 gene in SMA patients. Hum. Mutat..

[13] Monani U.R., Lorson C.L., Parsons D.W., Prior T.W., Androphy E.J., Burghes A.H., McPherson J.D. (1999). A single nucleotide difference that alters splicing patterns distinguishes the SMA gene SMN1 from the copy gene SMN2. Hum. Mol. Genet..

[14] Prior T.W., Nagan N., Sugarman E.A., Batish S.D., Braastad C. (2011). Technical standards and guidelines for spinal muscular atrophy testing. Genet. Med..

[15] Vitte J., Fassier C., Tiziano F.D., Dalard C., Soave S., Roblot N., Brahe C., Saugier-Veber P., Bonnefont J.P., Melki J. (2007). Refined characterization of the expression and stability of the SMN gene products. Am. J. Pathol..

[16] Soler-Botija C., Cuscó I., Caselles L., López E., Baiget M., Tizzano E.F. (2005). Implication of fetal SMN2 expression in type I SMA pathogenesis: Protection or pathological gain of function?. J. Neuropathol. Exp. Neurol..

[17] Boza-Morán M.G., Martínez-Hernández R., Bernal S., Wanisch K., Also-Rallo E., Le Heron A., Alías L., Denis C., Girard M., Yee J.-K. (2015). Decay in survival motor neuron and plastin 3 levels during differentiation of iPSC-derived human motor neurons. Sci. Rep..

[18] Wirth B., Garbes L., Riessland M. (2013). How genetic modifiers influence the phenotype of spinal muscular atrophy and suggest future therapeutic approaches. Curr. Opin. Genet. Dev..

[19] Calucho M., Bernal S., Alías L., March F., Venceslá A., Rodríguez-Álvarez F.J., Aller E., Fernández R.M., Borrego S., Millán J.M. (2018). Correlation between SMA type and SMN2 copy number revisited: An analysis of 625 unrelated Spanish patients and a compilation of 2834 reported cases. Neuromuscul. Disord..

[20] Costa-Roger M., Blasco-Pérez L., Cuscó I., Tizzano E.F. (2021). The Importance of Digging into the Genetics of SMN Genes in the Therapeutic Scenario of Spinal Muscular Atrophy. Int. J. Mol. Sci..

[21] Ruhno C., McGovern V.L., Avenarius M.R., Snyder P.J., Prior T.W., Nery F.C., Muhtaseb A., Roggenbuck J.S., Kissel J.T., Sansone V.A. (2019). Complete sequencing of the SMN2 gene in SMA patients detects SMN gene deletion junctions and variants in SMN2 that modify the SMA phenotype. Hum. Genet..

[22] Prior T.W., Krainer A.R., Hua Y., Swoboda K.J., Snyder P.C., Bridgeman S.J., Burghes A.H.M., Kissel J.T. (2009). A positive modifier of spinal muscular atrophy in the SMN2 gene. Am. J. Hum. Genet..

[23] Bernal S., Alías L., Barceló M.J., Also-Rallo E., Martínez-Hernández R., Gámez J., Guillén-Navarro E., Rosell J., Hernando I., Rodríguez-Alvarez F.J. (2010). The c.859G>C variant in the SMN2 gene is associated with types II and III SMA and originates from a common ancestor. J. Med. Genet..

[24] Wu X., Wang S.H., Sun J., Krainer A.R., Hua Y., Prior T.W. (2017). A-44G transition in SMN2 intron 6 protects patients with spinal muscular atrophy. Hum. Mol. Genet..

[25] Vezain M., Saugier-Veber P., Goina E., Touraine R., Manel V., Toutain A., Fehrenbach S., Frébourg T., Pagani F., Tosi M. (2010). A rare SMN2 variant in a previously unrecognized composite splicing regulatory element induces exon 7 inclusion and reduces the clinical severity of spinal muscular atrophy. Hum. Mutat..

[26] Karczewski K.J., Francioli L.C., Tiao G., Cummings B.B., Alföldi J., Wang Q., Collins R.L., Laricchia K.M., Ganna A., Birnbaum D.P. (2020). The mutational constraint spectrum quantified from variation in 141,456 humans. Nature.

[27] Glusman G., Caballero J., Mauldin D.E., Hood L., Roach J.C. (2011). Kaviar: An accessible system for testing SNV novelty. Bioinformatics.

[28] NHLBI and University of Michigan The NHLBI TransOmics for Precision Medicine (TOPMed) Whole Genome Sequencing Program. BRAVO Variant Browser (University of Michigan) 2018. https://bravo.sph.umich.edu/freeze5/hg38/.

[29] Jaganathan K., Panagiotopoulou S.K., McRae J.F., Darbandi S.F., Knowles D., Li Y.I., Kosmicki J.A., Arbelaez J., Cui W., Schwartz G.B. (2019). Predicting Splicing from Primary Sequence with Deep Learning. Cell.

[30] Ke S., Shang S., Kalachikov S.M., Morozova I., Yu L., Russo J.J., Ju J., Chasin L.A. (2011). Quantitative evaluation of all hexamers as exonic splicing elements. Genome Res..

[31] Souza P.V.S., Pinto W.B.V.R., Ricarte A., Badia B.M.L., Seneor D.D., Teixeira D.T., Caetano L., Gonçalves E.A., Chieia M.A.T., Farias I.B. (2021). Clinical and radiological profile of patients with spinal muscular atrophy type 4. Eur. J. Neurol..

[32] Wadman R.I., Jansen M.D., Stam M., Wijngaarde C.A., Curial C.A.D., Medic J., Sodaar P., Schouten J., Vijzelaar R., Lemmink H.H. (2020). Intragenic and structural variation in the SMN locus and clinical variability in spinal muscular atrophy. Brain Commun..

[33] Bowen B.M., Truty R., Aradhya S., Bristow S.L., Johnson B.A., Morales A., Tan C.A., Westbrook M.J., Winder T.L., Chavez J.C. (2021). SMA Identified: Clinical and Molecular Findings From a Sponsored Testing Program for Spinal Muscular Atrophy in More Than 2,000 Individuals. Front. Neurol..

[34] Pane M., Donati M.A., Cutrona C., De Sanctis R., Pirinu M., Coratti G., Ricci M., Palermo C., Berti B., Leone D. (2022). Neurological assessment of newborns with spinal muscular atrophy identified through neonatal screening. Eur. J. Pediatr..

[35] Cuscó I., Bernal S., Blasco-Pérez L., Calucho M., Alias L., Fuentes-Prior P., Tizzano E.F. (2020). Practical guidelines to manage discordant situations of SMN2 copy number in patients with spinal muscular atrophy. Neurol. Genet..

[36] Griffiths W.M., Miller A.J.F., Suzuki J.H., Lewontin D.T., Gelbart R. (2000). Chapter 14—Mutation, repair, and recombination. An introduction to Genetic Analysis.

[37] Alías L., Bernal S., Barceló M.J., Also-Rallo E., Martínez-Hernández R., Rodríguez-Alvarez F.J., Hernández-Chico C., Baiget M., Tizzano E.F. (2011). Accuracy of marker analysis, quantitative real-time polymerase chain reaction, and multiple ligation-dependent probe amplification to determine SMN2 copy number in patients with spinal muscular atrophy. Genet. Test. Mol. Biomark..

[38] Liu J., Huang S., Sun M., Liu S., Liu Y., Wang W., Zhang X., Wang H., Hua W. (2012). An improved allele-specific PCR primer design method for SNP marker analysis and its application. Plant Methods.

